# A bibliometric analysis of the 50 most cited articles about quality of life in patients with atrial fibrillation

**DOI:** 10.1186/s43044-025-00616-4

**Published:** 2025-02-17

**Authors:** Muhammad Arslan Ul Hassan, Sana Mushtaq, Tao Li, Zhen Yang

**Affiliations:** 1 Ningxia Medical University, Yinchuan, China; 2 Ningxia Medical University General Hospital, Yincham, China

**Keywords:** Atrial fibrillation, Quality of life, Bibliometric analysis

## Abstract

**Background:**

Atrial fibrillation is a progressive arrhythmia that has become a global concern affecting the quality of life of millions of patients. This bibliometric analysis aims to highlight the top 50 most cited articles in the field of quality of life in atrial fibrillation patients, focusing on publication trends, citations, influential authors and journals, promising institutions, and key contributing countries.

**Results:**

The top 50 articles were published between 1995 and 2021 across 15 journals, with an average number of citations of 380.5 per article. The article with the highest number of citations, i.e., 1525, was published in the ‘Circulation’ journal. ‘Natale Andrea’ and ‘Verma Atul’ contributed the most to the field, with 7 articles each. The United States of America had the highest total number of publications among the countries, with 20 articles. The field is extensively researched; however, there remains a necessity for methodological enhancements in the assessment of quality of life.

**Conclusions:**

This study assessed advancements in research regarding quality of life in atrial fibrillation patients and serves as an invaluable resource for clinicians and researchers to comprehend the existing body of knowledge in the field. Although quality of life in patients with atrial fibrillation has been the focus of interest in recent years, the management of patients still needs improvement.

## Background

Atrial fibrillation (AF) is a progressive arrhythmia that affects millions of people around the globe and presents major health challenges due to its associated complications, such as stroke, heart failure, and cognitive dysfunction. AF is characterized by an irregularly irregular heart rhythm, which can lead to debilitating symptoms and a notable reduction in quality of life (QoL) [1]. As healthcare providers recognized that the effects of AF extend beyond the presenting symptoms, the impact of AF on QoL became an important area of focus. Various factors influence QoL in patients with AF, including the severity and frequency of arrhythmic episodes; emotional and psychological burdens, such as anxiety and depression; and personal characteristics [2].

Many studies have explored the area of QoL in AF patients, and have tried to explain the factors that affect QoL, the ways of improving QoL, effective management strategies in AF patients, and so on [3–6]. Additionally, many specific methods have been developed to measure QoL in AF patients [7, 8]. Despite the growing literature on AF and its effects on QoL, comprehensive analyses of research trends remain sparse.

Since the publication of the first bibliometric analysis in the 1980s [9], it has become a valuable statistical tool for exploring research trends and patterns within specific domains [10]. By going through the published literature systematically, bibliometric analysis studies can uncover key hotspots, prolific authors, and influential journals and countries within a field. It not only focuses on research fields and the influence of scientific researchers and institutions, but also evaluates the impact of research outcomes. It can identify specific trends in scientific research and furnish evidence for scientific decision-making [11].

The last decade has seen many bibliometric analyses in the field of AF, but none of them have discussed QoL in patients with AF [12–15]. In the context of QoL in AF, a bibliometric analysis will reveal the current state of research and identify existing gaps, offering insights that can direct future research. Conducting a comprehensive bibliometric analysis of the top 50 most cited articles will also provide a unique lens through which one can examine the evolution of research focused on QoL among AF patients, highlighting shifts in research priorities and identifying areas ripe for further exploration. The choice to restrict the bibliometric analysis to the 50 most cited articles aims to concentrate on significant original research that has influenced the field. This approach ensures a manageable dataset that balances comprehensiveness and practicality while emphasizing the most impactful research.

This study aims to undertake a thorough bibliometric analysis to chart the development of research regarding QoL in patients with AF. Despite the extensive literature on QoL in AF, a bibliometric analysis can aid researchers by examining publication trends, citation networks, and thematic developments over time, thereby enhancing their understanding of the current status of QoL in AF. This bibliometric analysis can assist researchers and clinicians in identifying trends that could inspire future studies and advancements in clinical practice.

## Method

### Data source and search strategy

This study was exempt from approval by the institutional review board because it utilized publicly available data. The Science Citation Index Expanded database of the Web of Science Core Collection was used to collect and retrieve the data. It is one of the most comprehensive databases to date, offering complete citation details that make it superior to other databases, such as PubMed or Scopus, for conducting bibliometric analyses. A ‘topic search’ in advance mode was used for the search query, and the search was conducted repeatedly employing different Boolean operators (AND, OR) to encompass all iterations. The keyword strategy consisted of [TS = (atrial fibrillation OR paroxysmal atrial fibrillation OR persistent atrial fibrillation OR auricular fibrillation) AND (quality of life OR QoL OR health-related quality of life)]. The search was restricted to articles written in English, the document type was article, and the date range was set from inception until October 2024.

This process yielded 97,949 results, which were then sorted according to the highest number of citations. The articles were subsequently assessed by title, abstract, keywords, and full text (if needed) for inclusion, selecting only those that contained both main keywords (AF and QoL) and discussing QoL in AF. Only original research studies, such as randomized control trials (RCTs), cohort studies, and case–control studies, were included. Articles that did not discuss QoL in AF patients were excluded. Review articles, abstracts, guidelines, consensus statements, scientific statements, systematic reviews, and meta-analyses were also excluded from the 50 most highly cited articles.

### Data extraction and analysis

All the steps were performed by two authors (MAUH and SM) independently, and disagreements on an article for selection were addressed through consensus or by consulting the senior author (ZY). The search was completed independently by both authors within one day on October 01, 2024. Fifty articles satisfied the inclusion criteria following the assessment of the top 1997 out of 97,949 search results. The complete manuscripts were acquired and examined for each of the 50 articles to extract the data. The level of evidence (LOE) was assessed for all 50 included articles to determine the relative risk of bias. The Oxford Centre for Evidence-Based Medicine (CEBM) guidelines were used by both authors independently to determine the LOE. The citation density for each article, which accounts for the impact of the article in years since publication, was also calculated. The citation density was calculated via the following equation:

Citation Density = Total citations to date / Number of years since publication.

The analysis in this study utilized R (Version 4.4.1) and the bibliometrix package, with the integration of “biblioshiny” into RStudio (Version 2024.09.0 + 375), thereby facilitating a comprehensive bibliometric analysis [16]. VOSviewer (version 1.6.20) was used for bibliographic coupling data. Correlation analysis was conducted as well utilizing Statistical package for the social sciences (SPSS for Windows, version 27, IBM corp., Armonk, NY, USA). The Spearman correlation coefficient (r) was utilized to assess the strength of correlations: high (r > 0.60), moderate (0.30 < r < 0.60), and weak (r < 0.30) with a significance level of α = 0.05.

## Results

A total of 97,949 articles were identified on the basis of our search strategy for QoL in AF patients. After going through the first 1997 articles with the highest number of citations, we were able to identify the top 50 articles. Table 1 shows the details of all 50 selected articles.

**Table 1:** All 50 of the most cited articles on QoL in patients with AF

Rank No	Article title	Publication year	Journal name	Journal impact factor (2023)	Total citations	Citations density
1	Lifetime risk for development of atrial fibrillation—The Framingham Heart Study	2004	Circulation	35.5	1525	72.6
2	Comparison of Antiarrhythmic Drug Therapy and Radiofrequency Catheter Ablation in Patients With Paroxysmal Atrial Fibrillation A Randomized Controlled Trial	2010	JAMA-Journal of the American medical associatiON	63.1	866	57.7
3	Rhythm or rate control in atrial fibrillation—Pharmacological intervention in atrial fibrillation (PIAF): a randomized trial	2000	Lancet	98.4	841	33.7
4	Radiofrequency ablation vs antiarrhythmic drugs as first-line treatment of symptomatic atrial fibrillation—A randomized trial	2005	JAMA-Journal of the American medical association	63.1	811	40.6
5	Randomized trial of rate control versus rhythm control in persistent atrial fibrillation—The Strategies of Treatment of Atrial Fibrillation (STAF) study	2003	Journal of the American college OF cardiology	21.7	682	31
6	Catheter ablation for atrial fibrillation in congestive heart failure	2004	New England journal of medicine	96.2	631	25.3
7	Catheter Ablation Versus Antiarrhythmic Drugs for Atrial Fibrillation The A4 Study	2008	Circulation	35.5	609	35.8
8	Mortality, morbidity, and quality of life after circumferential pulmonary vein ablation for atrial fibrillation—Outcomes from a controlled nonrandomized long-term study	2003	Journal of the American college of cardiology	21.7	588	26.7
9	Effect of Weight Reduction and Cardiometabolic Risk Factor Management on Symptom Burden and Severity in Patients With Atrial Fibrillation A Randomized Clinical Trial	2013	JAMA-Journal of the American medical association	63.1	583	48.6
10	Ablation Versus Amiodarone for Treatment of Persistent Atrial Fibrillation in Patients With Congestive Heart Failure and an Implanted Device: Results From the AATAC Multicenter Randomized Trial	2016	Circulation	35.5	565	62.8
11	Amiodarone versus sotalol for atrial fibrillation	2005	New England journal of medicine	96.2	538	26.9
12	Pulmonary vein isolation for atrial fibrillation in patients with heart failure	2008	New England journal of medicine	96.2	505	29.7
13	Radiofrequency Ablation vs Antiarrhythmic Drugs as First-Line Treatment of Paroxysmal Atrial Fibrillation (RAAFT-2) A Randomized Trial	2014	JAMA-Journal of the American medical association	63.1	451	41
14	The impairment of health-related quality of life in patients with intermittent atrial fibrillation: Implications for the assessment of investigational therapy	2000	JOURNAL OF THE AMERICAN COLLEGE OF CARDIOLOGY	21.7	450	18
15	Cryoablation or Drug Therapy for Initial Treatment of Atrial Fibrillation	2021	New England journal of medicine	96.2	437	109.3
16	Effect of Catheter Ablation vs Medical Therapy on Quality of Life Among Patients With Atrial Fibrillation The CABANA Randomized Clinical Trial	2019	JAMA-Journal of the American medical association	63.1	379	63.2
17	Long-term risk of recurrent atrial fibrillation as documented by an Implantable monitoring device -: Implications for optimal patient care	2004	Journal of the American college of cardiology	21.7	367	17.5
18	Perception of atrial fibrillation before and after radiofrequency catheter ablation—Relevance of asymptomatic arrhythmia recurrence	2005	Circulation	35.5	360	14.4
19	Impact of CARDIOrespiratory FITness on Arrhythmia Recurrence in Obese Individuals With Atrial Fibrillation The CARDIO-FIT Study	2015	Journal of the American college of cardiology	21.7	355	35.5
20	Effect of rate or rhythm control on quality of life in persistent atrial fibrillation—Results from the Rate Control Versus Electrical Cardioversion (RACE) study	2004	Journal of the American college OF cardiology	21.7	342	16.3
21	A Randomized Trial to Assess Catheter Ablation Versus Rate Control in the Management of Persistent Atrial Fibrillation in Heart Failure	2013	Journal of the American college of cardiology	21.7	339	28.3
22	Development and Validation of the Atrial Fibrillation Effect on QualiTy-of-Life (AFEQT) Questionnaire in Patients With Atrial Fibrillation	2011	Circulation-arrhythmia and electrophysiology	9.1	315	22.5
23	Comparative effects of permanent biventricular and right-univentricular pacing in heart failure patients with chronic atrial fibrillation	2002	European heart journal	37.6	313	13.6
24	The effect of stroke and stroke prophylaxis with aspirin or warfarin on quality of life	1996	Archives of internal medicine	22.5	312	10.8
25	Gender-related differences in presentation, treatment, and outcome of patients with atrial fibrillation in Europe -: A report from the Euro Heart Survey on atrial fibrillation	2007	Journal of the American college of cardiology	21.7	302	16.8

26	Cardiac resynchronization in patients with congestive heart failure and chronic atrial fibrillation—Effect of upgrading to biventricular pacing after chronic right ventricular pacing	2002	Journal of the American college of cardiology	21.7	283	12.3
27	Cost-effectiveness of warfarin and aspirin for prophylaxis of stroke in patients with nonvalvular atrial-fibrillation	1995	JAMA-Journal of the american medical association	63.1	280	9.3
28	Progression to chronic atrial fibrillation after the initial diagnosis of paroxysmal atrial-fibrillation: Results from the Canadian Registry of Atrial Fibrillation	2005	American heart journal	3.7	270	13.5
29	Four-year efficacy of cardiac resynchronization therapy on exercise tolerance and disease progression—The importance of performing atrioventricular junction ablation in patients with atrial fibrillation	2006	Journal of the American college of cardiology	21.7	267	14.1
30	Ablation Versus Drug Therapy for Atrial Fibrillation in Heart Failure Results From the CABANA Trial	2021	Circulation	35.5	264	66
31	Catheter ablation vs. antiarrhythmic drug treatment of persistent atrial fibrillation: a multicentre, randomized, controlled trial (SARA study)	2014	European heart journal	37.6	261	23.7
32	Atrial fibrillation in stroke-free patients is associated with memory impairment and hippocampal atrophy	2008	European heart journal	37.6	249	14.7
33	Pulmonary vein isolation for the treatment of atrial fibrillation in patients with impaired systolic function	2004	Journal of the American college of cardiology	21.7	249	11.9
34	Radiofrequency ablation for persistent atrial fibrillation in patients with advanced heart failure and severe left ventricular systolic dysfunction: a randomized controlled trial	2011	Heart	5.1	247	17.6
35	Assessment of atrioventricular junction ablation and VVIR pacemaker versus pharmacological treatment in patients with heart failure and chronic atrial fibrillation—A randomized, controlled study	1998	Circulation	35.5	243	9
36	Cryoballoon or radiofrequency ablation for symptomatic paroxysmal atrial fibrillation: reintervention, rehospitalization, and quality-of-life outcomes in the FIRE AND ICE trial	2016	European heart journal	37.6	239	26.6
37	Discerning the Incidence of Symptomatic and Asymptomatic Episodes of Atrial Fibrillation Before and After Catheter Ablation (DISCERN AF) A Prospective, Multicenter Study	2013	JAMA internal medicine	22.5	233	19.4
38	Cost-Effectiveness of Using Pharmacogenetic Information in Warfarin Dosing for Patients With Nonvalvular Atrial Fibrillation	2009	Annals of internal medicine	19.6	227	14.2
39	Depression, anxiety, and quality of life in patients with atrial fibrillation	2007	Chest	9.5	213	11.8
40	The ablate and pace trial: A prospective study of catheter ablation of the AV conduction system and permanent pacemaker implantation for treatment of atrial fibrillation	1998	Journal of interventional cardiac electrophysiology	2.1	205	7.6
41	Effect of Catheter Ablation vs Antiarrhythmic Medication on Quality of Life in Patients With Atrial Fibrillation The CAPTAF Randomized Clinical Trial	2019	JAMA-Journal of the American medical association	63.1	202	33.7
42	Prevention of ventricular desynchronization by permanent para-Hisian pacing after atrioventricular node ablation in chronic atrial fibrillation	2006	Journal of the American college of cardiology	21.7	192	10.1
43	Quality of life improves with treatment in the Canadian Trial of Atrial Fibrillation	2002	American heart journal	3.7	192	8.4
44	Quality of life in atrial fibrillation: The Atrial Fibrillation Follow-up Investigation of Rhythm Management (AFFIRM) study	2005	American heart journal	3.7	191	9.6
45	Clinical Profile and Consequences of Atrial Fibrillation in Hypertrophic Cardiomyopathy	2017	Circulation	35.5	186	23.3
46	Long-Term Quality of Life After Ablation of Atrial Fibrillation The Impact of Recurrence, Symptom Relief, and Placebo Effect	2010	Journal of the American college of cardiology	21.7	176	11.7
47	Quality of life and exercise performance in patients in sinus rhythm versus persistent atrial fibrillation—A veterans affairs cooperative studies program substudy	2006	Journal of the American college of cardiology	21.7	176	9.3
48	Aerobic Interval Training Reduces the Burden of Atrial Fibrillation in the Short Term A Randomized Trial	2016	Circulation	35.5	172	19.1
49	Association Between Atrial Fibrillation Symptoms, Quality of Life, and Patient Outcomes Results From the Outcomes Registry for Better Informed Treatment of Atrial Fibrillation (ORBIT-AF)	2015	Circulation-cardiovascular quality and outcomes	6.2	172	17.2
50	Assessment of atrioventricular junction ablation and DDDR mode-switching pacemaker versus pharmacological treatment in patients with severely symptomatic paroxysmal atrial fibrillation—A randomized controlled study	1997	Circulation	35.5	171	6.1

### Publication timeline:

**Fig. 1:**
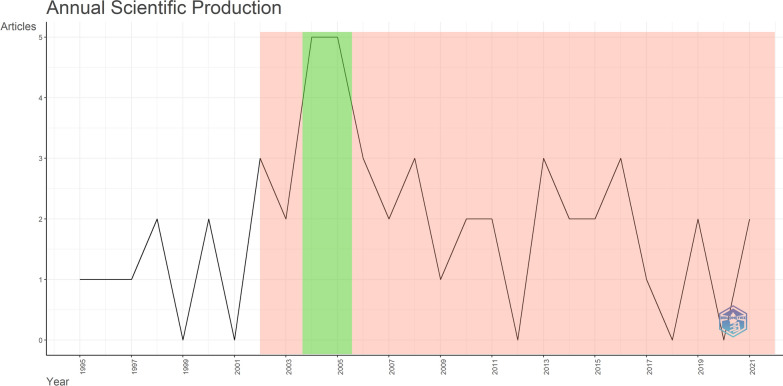
The distribution of publications by year is illustrated, with the red area indicating the period during which the majority of articles were published. The green area highlights the years with the highest number of publications

The publication timeline of the 50 most highly cited articles on QoL in AF patients showed a fluctuating trend (Fig. 1). The most recent article included in our analysis was published in 2021, whereas the earliest article was published in 1995. The majority of the articles were published after 2002 (43 out of 50), and half (25) of the articles were published after 2007. The years with the most publications were 2004 and 2005, with 5 publications each. The article with the highest number of citations among the 50 selected articles was published in 2004 (1525 citations), and the article with the lowest number of citations was published in 1997 (171 citations). A Spearman correlation analysis was performed to investigate the relationship between publication year and citation count. Results indicated a weak negative correlation (*r*_*s*_ = -0.116, *p* < 0.05), suggesting that older publications generally exhibit slightly higher citation counts than more recent publications.

### Citation analysis

The 50 most cited articles on QoL in AF patients accumulated a total of 19,026 citations up to the date of our analysis, with a mean number of citations of 380.5 per article. The highest number of citations of a single article was 1525, whereas the lowest number of citations was 171. The median number of citations was 301.3, with a standard deviation of 144.7. The peak of total mean citations per article was noted in 2000, with 645.5 citations. The two most recent articles from 2021 ranked 15th and 30th, with total citations of 437 and 264, respectively. On the other hand, the earliest published article from 1995 ranked 27th with a total of 280 citations. The highest citation density of an article was 109.3; the lowest citation density was 6.1.

**Fig. 2:**
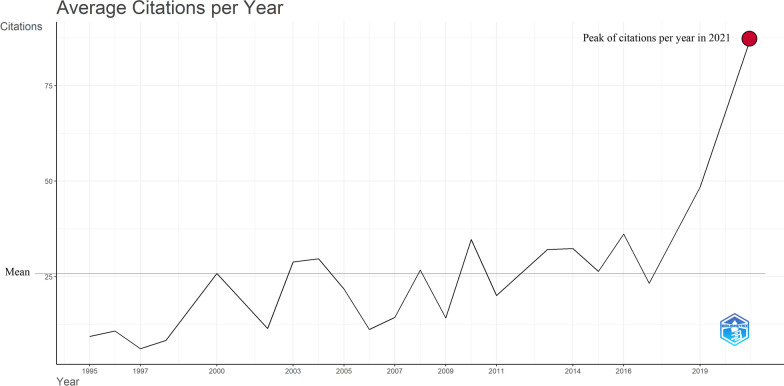
The distribution of citations for the 50 most cited articles

**Fig. 3:**
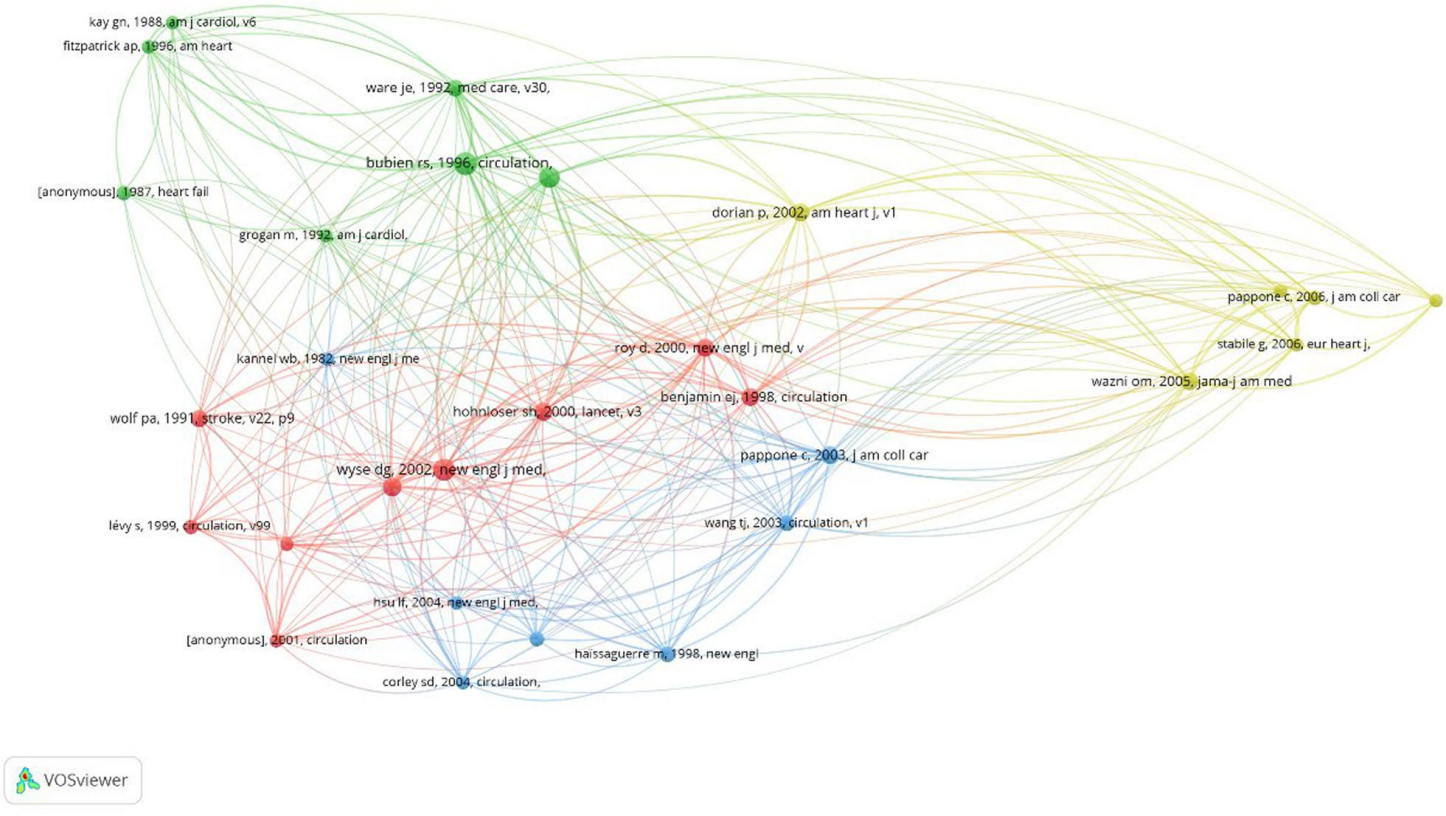
Co-citation network analysis of the top 50 articles. The minimum number of citations per article is five

The mean number of citations per year was 25.4. In 2021, the total mean citations per year reached a peak of 87.6. Figure 2 elaborates the average number of citations acquired by the 50 most cited articles per year. Additionally, the co-citation network map (Fig. 3) identified four unique clusters, each signifying important topics within the research domain. The red cluster comprised foundational works, the green cluster emphasized methodological breakthroughs, the yellow cluster covered emergent research, and the blue cluster focused on specialty themes.

A Spearman correlation analysis was performed to evaluate the relationship between journal impact factor and citation counts. The results indicated a moderate positive correlation (*r*_*s*_ = 0.43, *p* < 0.05), suggesting that journals with higher impact factors are associated with increased citation counts.

### Analysis of authors and journals

**Fig. 4:**
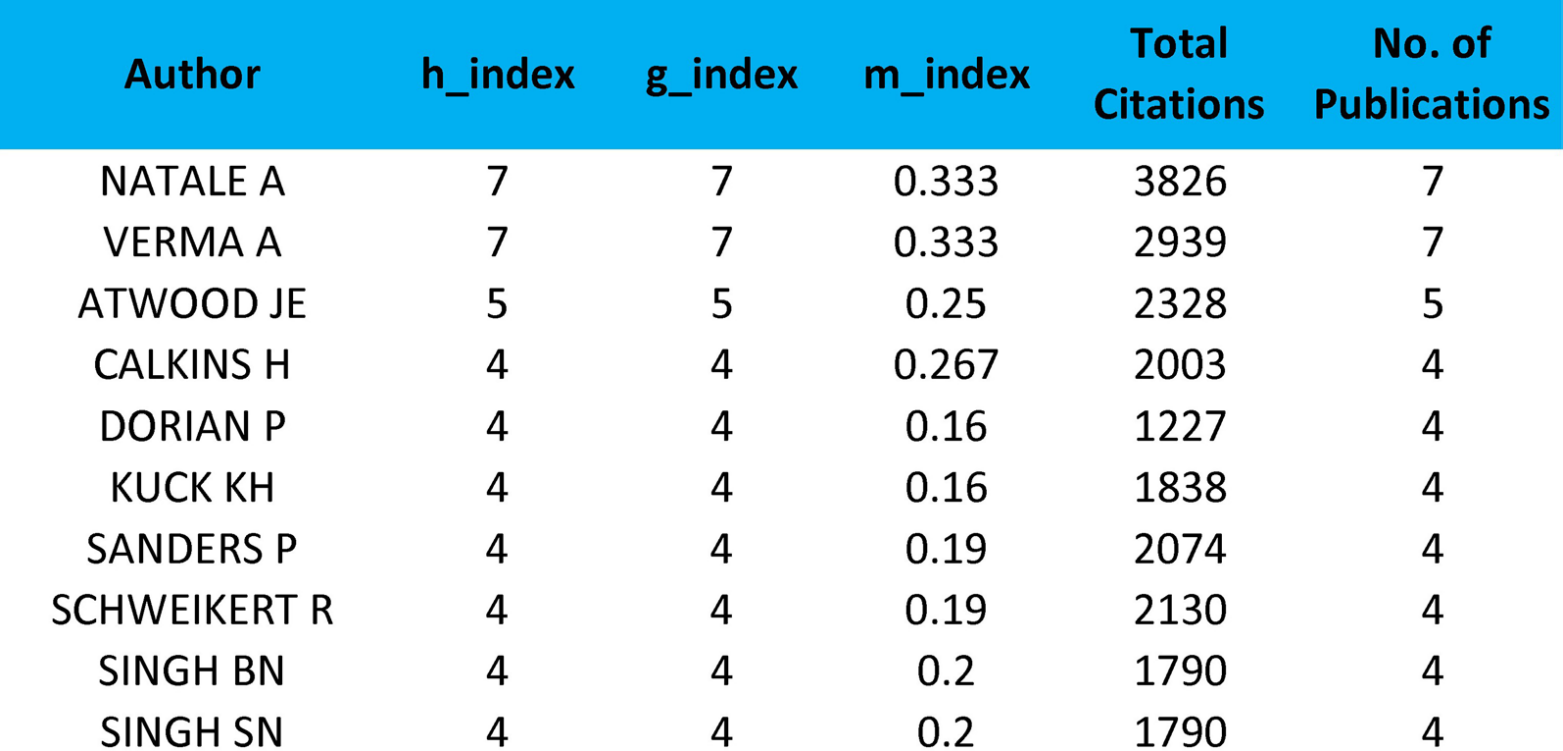
Top ten authors with the highest number of citations and publications

**Fig. 5:**
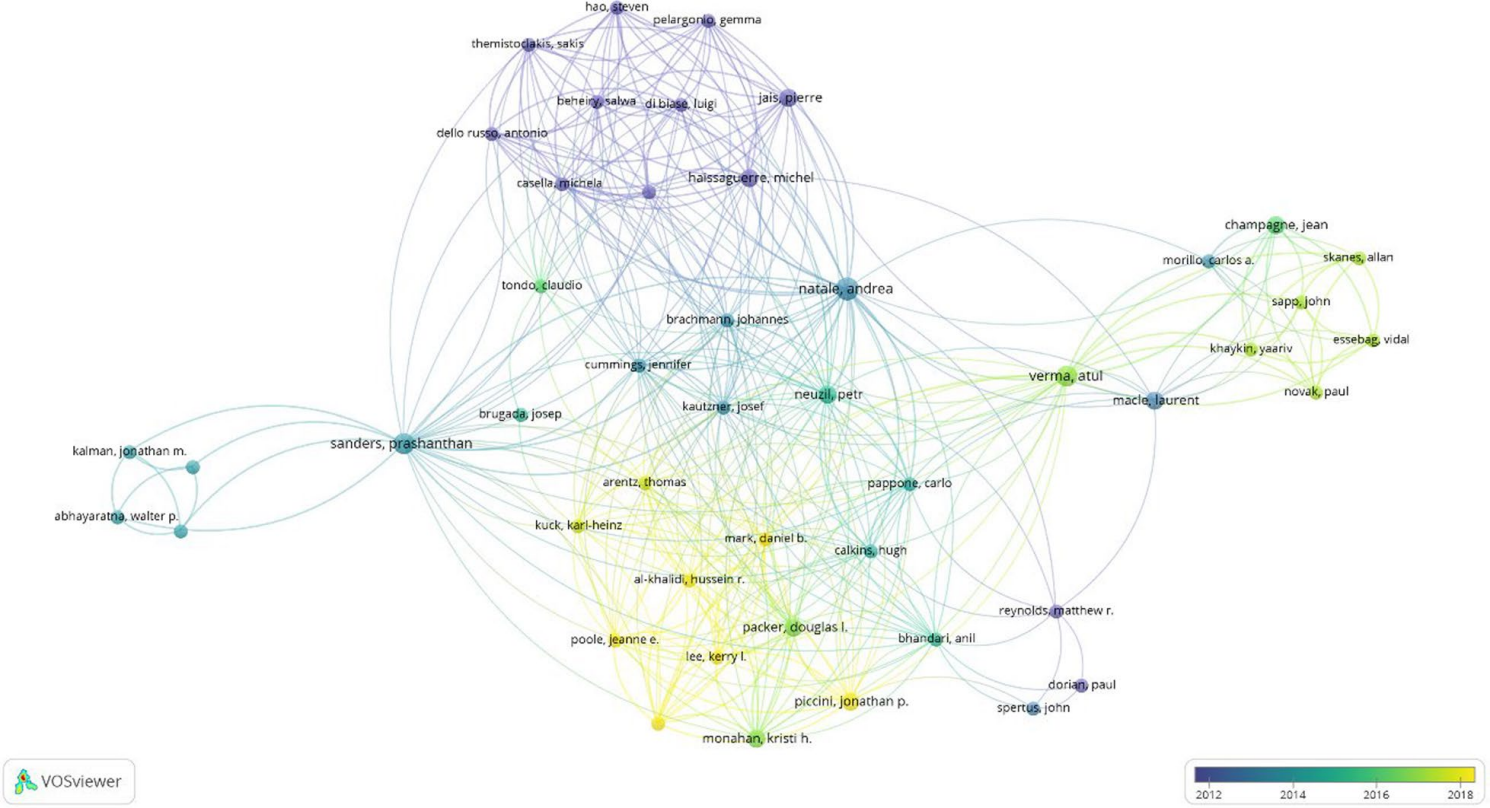
Co-authorship network map among the top 50 articles. Minimum number of occurrences of the author: 2 articles

Among the 50 most cited articles, the authors who had the leading contribution in the field of QoL in AF were “Natale Andrea” and “Verma Atul”, with contributions of 7 articles each, with 3826 and 2939 citations, respectively. The third on the list was “Atwood JE”, with 5 contributions and a total of 2328 citations. The impact of the top 10 authors among a total of 877 authors is shown in Fig. 4. The average number of co-authors per article was 20.7. Furthermore, Fig. 5 illustrates their collaborative networks and interactions within the field, offering a detailed overview of their scholarly connections and collaborative efforts. The analyses highlight the significant roles of individual authors and their collaborative networks in promoting knowledge and innovation within the field.

**Fig. 6:**
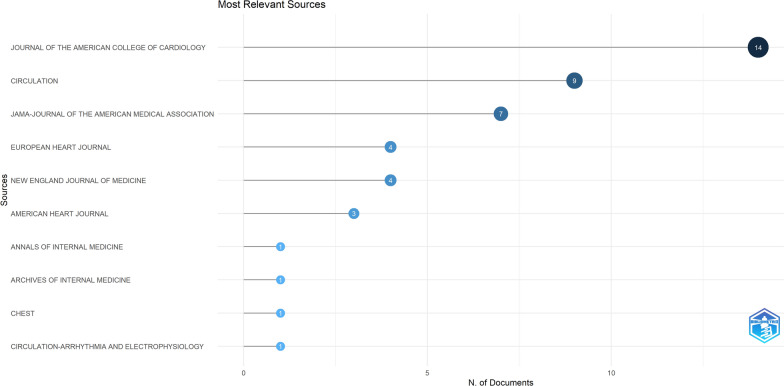
Top 10 journals with the most publications

The journals with the highest impact and leading contribution in this field numbered 15 in total. The ‘Journal of the American College of Cardiology’ was the leading journal, with 14 articles, and ‘Circulation’ was second on the list, with 9 articles. Other notable journals include the Journal of the American Medical Association, European Heart Journal, and New England Journal of Medicine. Figure 6 illustrates the top 10 journals, highlighting their dominance in the field of QoL in AF. The journal with the highest impact factor of 98.4 was “Lancet,” which published only one article in this field, followed by “New England Journal of Medicine”, with an impact factor of 96.2, which published four articles, according to the 2023 JCR year.

### Institutions and countries analysis

**Fig. 7:**
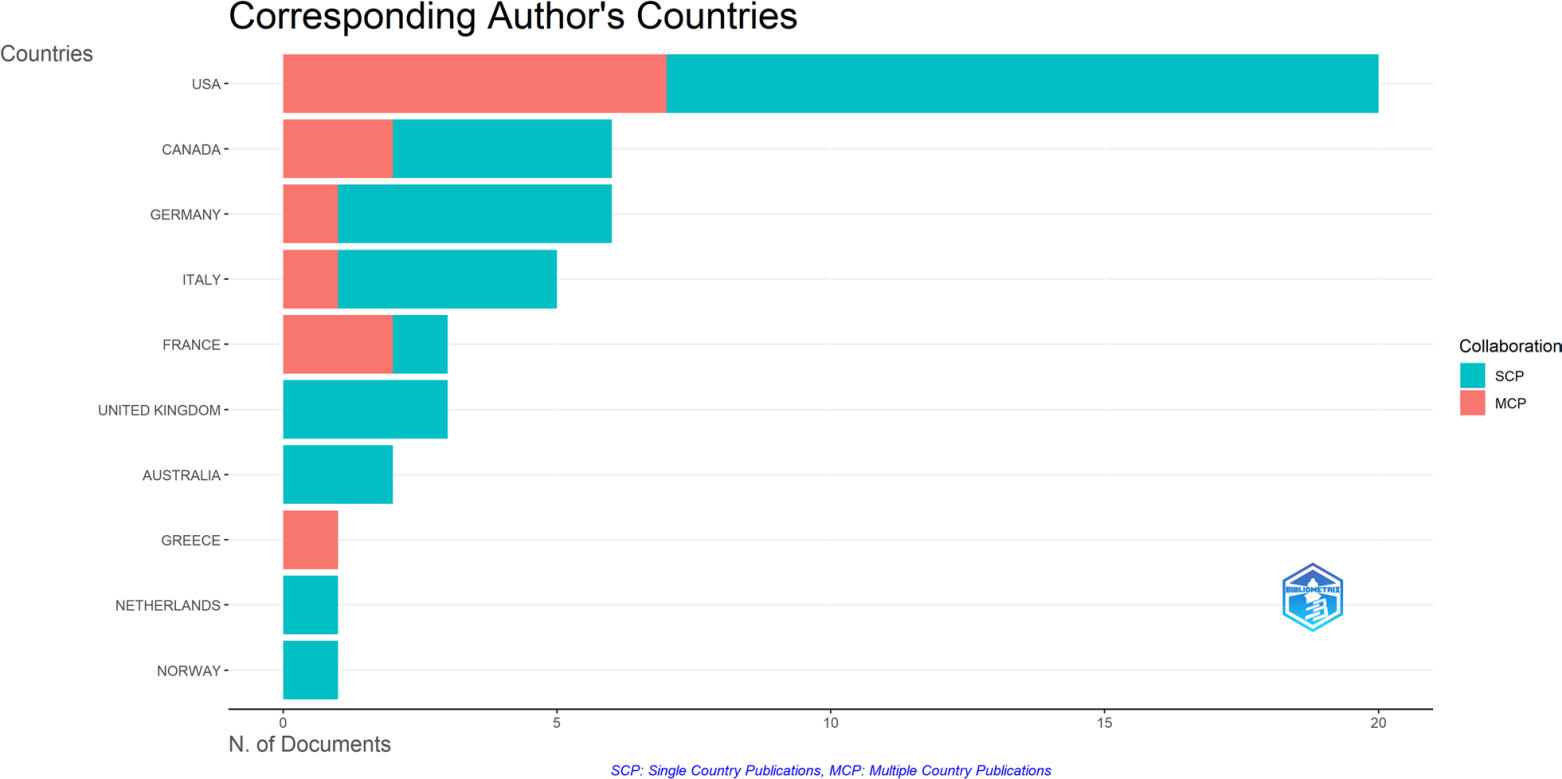
Top 10 countries with the most publications

The pertinent institutions contributing to research on QoL in AF patients were also identified, highlighting institutions that have made substantial contributions to this specialized field. The ‘West Los Angeles Veterans Affairs Medical Center’ and ‘Stanford University’ ranked first and second with 14 and 8 articles, respectively. Other notable institutions include Duke University, the University of Milan, and the University of Münster. On the other hand, the United States of America had the highest number of publications, totaling 20 articles, followed by Canada and Germany, with 6 articles each, and Italy with 5 articles. The details of the top 10 countries are given in Fig. 7, which shows both multiple country publications and single country publications.

### Analysis of studies and keywords

**Fig. 8:**
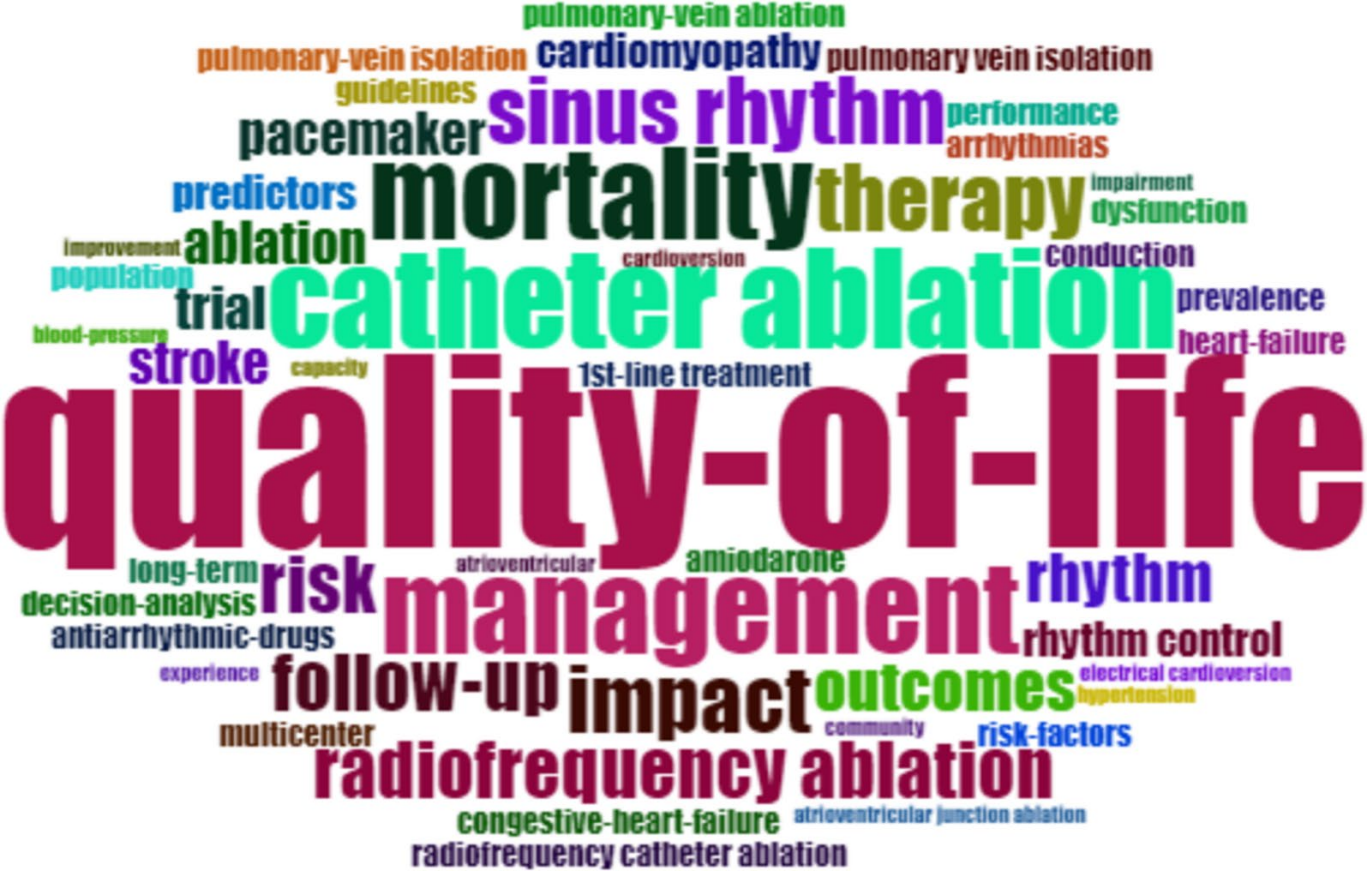
Most commonly used keywords among the top 50 highly cited articles

**Fig. 9:**
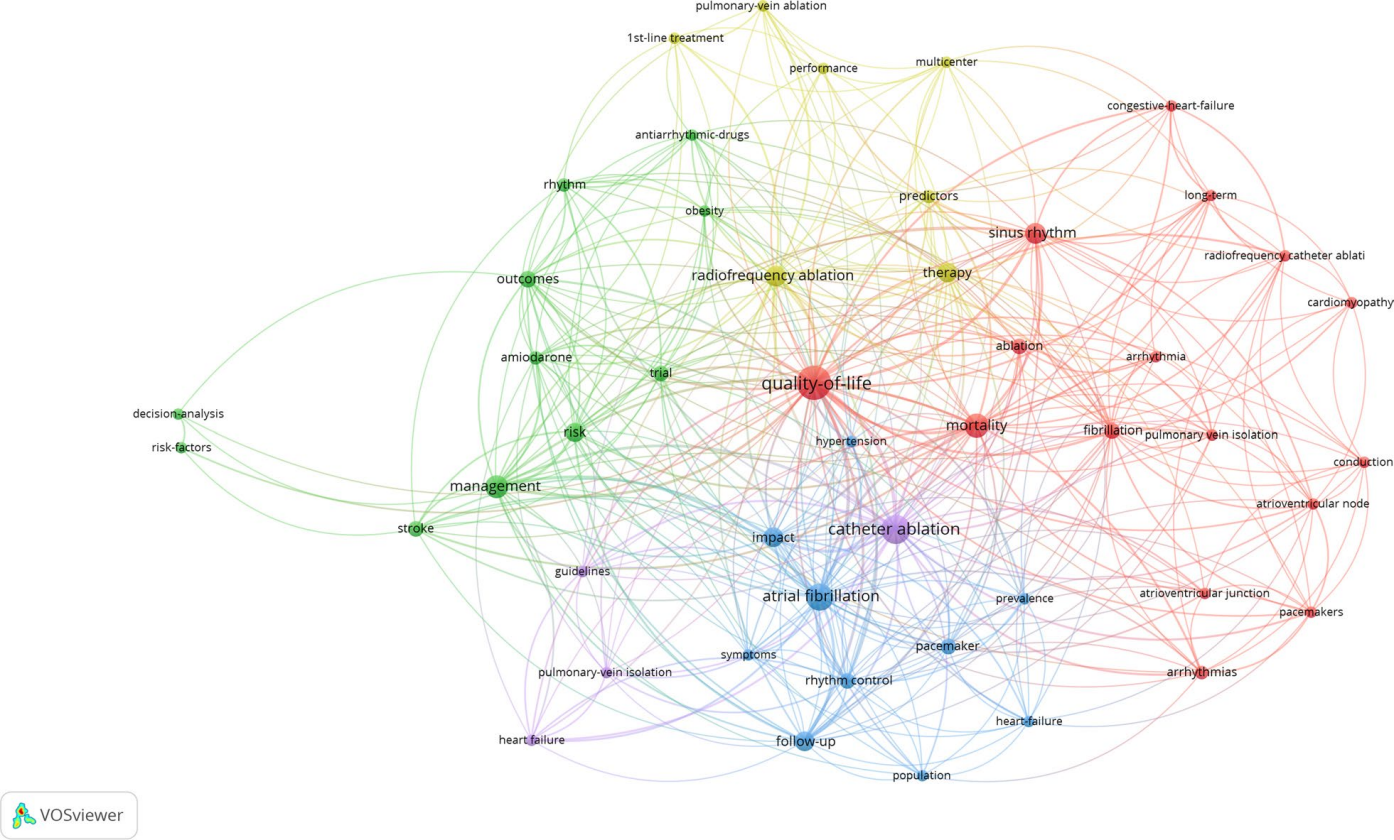
Network map of the most common keywords of the top 50 articles. Minimum number of keywords: 3 keywords

The most prevalent study type among the top 50 most cited articles was randomized control trials, followed by cohort studies and case series. Furthermore, the most common keyword used in these 50 highly cited articles was ‘QoL’ followed by AF, catheter ablation, management, mortality, impact, sinus rhythm, and so on. Figure 8 displays a word cloud of the keywords, while Fig. 9 presents a network map of the most prevalent keywords.

### Collaboration analysis

**Fig. 10:**
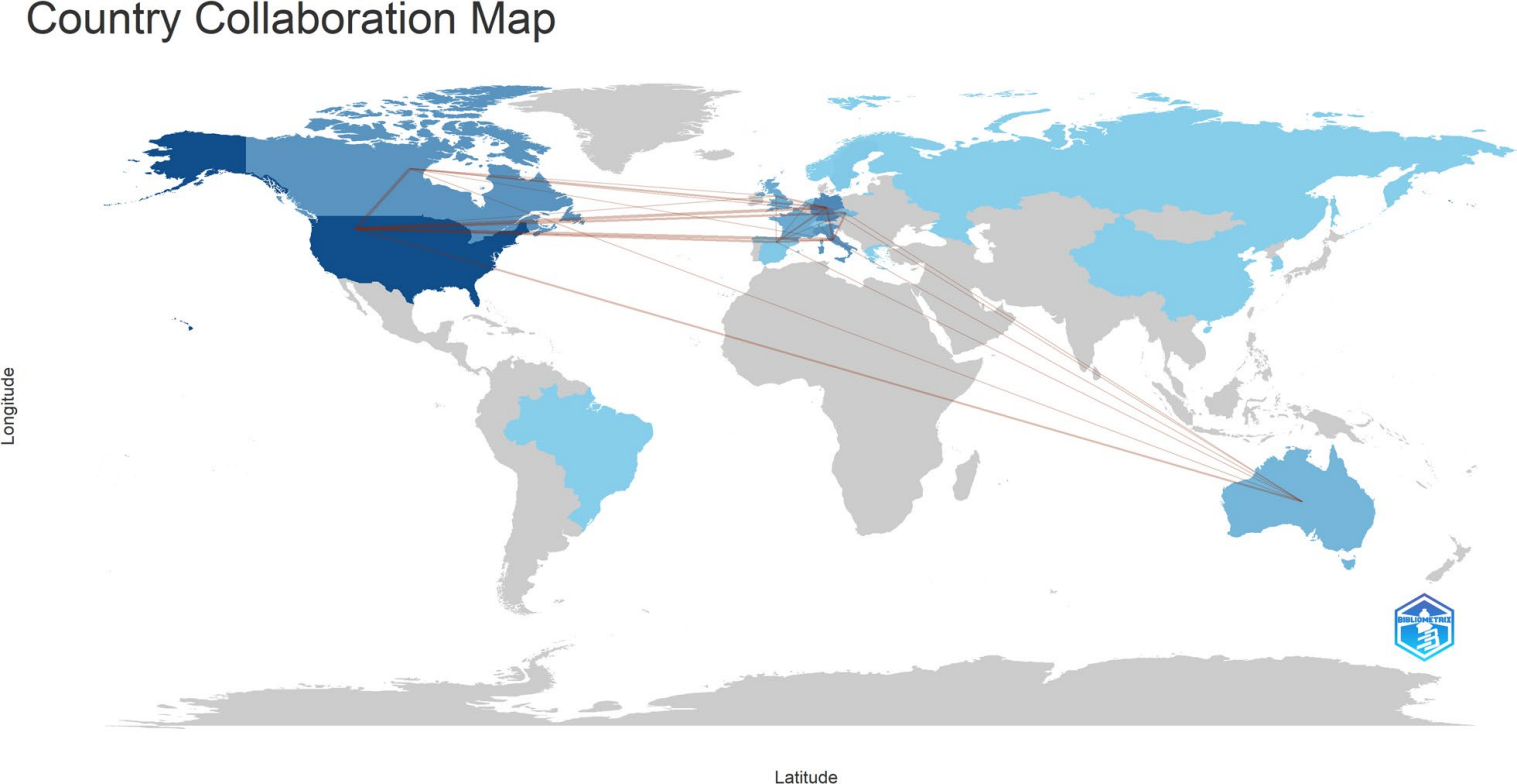
Collaboration map of countries with the 50 most-cited articles

The international collaboration on studies regarding QoL in patients with AF revealed a diverse and interconnected landscape. The broad collaborations between United States of America – Canada, United States of America – Germany, United States of America – Italy, and Germany – Italy highlighted the international cooperation between countries in the specific field. Figure 10 visually depicts the global spread and connections of these collaborations, highlighting the influence of international partnerships on the field’s academic dialog.

## Discussion

Treatment strategies to improve the QoL of AF patients and accurately measure QoL in AF patients remain topics of interest for researchers. Through a bibliometric review of QoL in AF, we identified the most significant and influential publications in this field. Our analysis equips clinicians managing AF with a tool to swiftly identify highly cited articles, eliminating the need to sift through extensive literature. Although the index included publication in our study taking place in 1995, the majority of highly cited articles were published after 2002. The 50 most cited articles in our study accumulated a total of 19,026 citations up to the date of our study, and the average number of citations increased incrementally after 2018.

Our analysis revealed a weak negative correlation between the number of citations of an article and its publication year, indicating that older articles generally receive more citations than newer ones. Additionally, a moderate positive correlation was observed between journal impact factor and citation count, suggesting that journals with higher impact factors attract greater researcher interest and, consequently, more citations. Furthermore, the increase in average citations post-2018 suggests a preference among researchers for reading and citing recent articles. This analysis may assist researchers and clinicians in identifying shifting priorities, new interventions, and overlooked areas, ensuring that clinical research addresses existing gaps and that treatment guidelines are based on the most reliable and current evidence. This approach promotes evidence-based improvements, enhancing patient outcomes and aligning care strategies with contemporary knowledge.

A total of 877 authors from 12 countries contributed to the 50 most cited articles in the field of QoL in AF. Forty percent of the articles, specifically 20 out of 50, were authored by individuals from the United States of America, indicating the country’s significant contribution to the field. This dominance can be attributed to the country’s robust research infrastructure, which supports and encourages professionals. Additionally, the country’s significant funding in medical research, a comprehensive network of leading research institutions (like the Stanford University), and a strong regulatory framework, exemplified by the Food and Drug Administration, may have contributed to its dominance in the field [17].

Our analysis revealed that 54% of the studies were of level I evidence, while 36% were of level II evidence, in accordance with CEBM guidelines. This suggests a preference among researchers for citing highly reliable studies, such as RCTs and cohort studies. Furthermore, it indicates a significant presence of high-quality level I and II evidence on this topic within the literature. The predominant study type identified was RCTs, with a primary focus on comparing antiarrhythmic drugs (AADs) and catheter ablation to determine which treatment option yields superior outcomes and improves QoL. Despite clinical guidelines recommending AADs as the first-line treatment for patients with AF, numerous studies advocate for catheter ablation as the preferred initial treatment approach [4, 18].

Although the comparison between catheter ablation and AADs has been thoroughly investigated, additional research is required to assess the effects of different ablation techniques on the QoL of patients with AF and the overall outcomes of AF treatment. The variability in QoL assessment tools utilized in AF research complicates study comparisons and may restrict the generalizability of findings across diverse populations. Therefore, the development and validation of disease-specific QoL assessment tools is crucial.

A further area for investigation is the duration from the diagnosis of AF to ablation and the impact of delayed ablation on the QoL of AF patients. Furthermore, to what degree does early intervention enhances QoL? Ablation strategies and approaches for patients with AF recurrence and durable pulmonary vein isolation represent another identified domain [19]. Additionally, the comparison of catheter ablation and medical therapy for the treatment of heart failure patients with AF warrants further investigation [20].

The field was contributed to by fifteen journals, with the highest impact factor being 98.4 (Lancet). The Journal of the American College of Cardiology was found to be leading this field, with 14 publications. No correlation existed between the number of citations of an article and the quantity of articles published by a journal. On the other hand, the dominance of the United States of America in the field continued, with five out of the top ten affiliations originating from the country.

While many bibliometric analyses have investigated trends and hotspots in various domains related to AF, this is the first bibliometric analysis focusing on the critical area of QoL for patients with AF. However, there are many limitations in our study that need to be acknowledged. First, our study had a narrow inclusion criterion and included only original articles, excluding reviews, meta-analyses, expert opinions, and clinical guidelines. The primary reason for the exclusion of these articles was their tendency to receive greater attention and, consequently, higher citation rates, potentially overshadowing the significance of other important articles. Second, we investigated only the 50 most cited peer-reviewed publications. This may lead to the underrepresentation of certain significant findings or recent advancements in the field of QoL in AF patients. Third, only citation counts were utilized as a metric, potentially introducing bias toward older studies that typically receive more citations due to their age. This may overshadow more recent, yet significant research that has not had sufficient time to accumulate citations. Fourth, the potential influence of the “Matthew effect”, as highly cited articles tend to maintain higher citation frequency over time, irrespective of their present relevance or quality. To mitigate these limitations, we used citation density metrics to account for the relative impact of older and newer studies and reported the LOE for the included studies, providing insight into both their scientific quality and citation influence. Finally, variations in citation practices across different fields and geographic regions may introduce bias, potentially distorting the representation of influential studies. Areas with higher citation rates may disproportionately highlight specific articles, potentially skewing the perceived influence of research.

## Conclusion

This study identifies the 50 most cited articles on QoL in patients with AF, which have garnered over 19,000 citations. The majority of the available research on the subject consisted of RCTs (54%), with all but seven of the most significant publications published between 2002 and 2021. The United States of America was the primary contributor, succeeded by Canada, Germany, and Italy. Researchers can access the most important articles on QoL in AF patients in the current literature with the aid of this bibliometric analysis.

## Data Availability

No datasets were generated or analyzed during the current study.

## Abbreviations

AF: Atrial Fibrillation
QoL: Quality of Life
LOE: Level of Evidence
CEBM: Centre for Evidence-Based Medicine
RCTs: Randomized Control Trials
AADs: Antiarrhythmic Drugs
